# The Hurdle Approach–A Holistic Concept for Controlling Food Safety Risks Associated With Pathogenic Bacterial Contamination of Leafy Green Vegetables. A Review

**DOI:** 10.3389/fmicb.2018.01965

**Published:** 2018-08-24

**Authors:** Lars Mogren, Sofia Windstam, Sofia Boqvist, Ivar Vågsholm, Karin Söderqvist, Anna K. Rosberg, Julia Lindén, Emina Mulaosmanovic, Maria Karlsson, Elisabeth Uhlig, Åsa Håkansson, Beatrix Alsanius

**Affiliations:** ^1^Microbial Horticulture, Department of Biosystems and Technology, Swedish University of Agricultural Sciences, Alnarp, Sweden; ^2^Department of Biological Sciences, SUNY Oswego, Oswego, NY, United States; ^3^Department of Biomedical Sciences and Veterinary Public Health, Swedish University of Agricultural Sciences, Alnarp, Sweden; ^4^Department of Food Technology, Engineering and Nutrition, Lund University, Lund, Sweden

**Keywords:** *Escherichia coli*, foodborne disease, food system, *listeria*, primary production processing, *Salmonella*, spoilage

## Abstract

Consumers appreciate leafy green vegetables such as baby leaves for their convenience and wholesomeness and for adding a variety of tastes and colors to their plate. In Western cuisine, leafy green vegetables are usually eaten fresh and raw, with no step in the long chain from seed to consumption where potentially harmful microorganisms could be completely eliminated, e.g., through heating. A concerning trend in recent years is disease outbreaks caused by various leafy vegetable crops and one of the most important foodborne pathogens in this context is Shiga toxin-producing *Escherichia coli* (STEC). Other pathogens such as *Salmonella, Shigella, Yersinia enterocolitica* and *Listeria monocytogenes* should also be considered in disease risk analysis, as they have been implicated in outbreaks associated with leafy greens. These pathogens may enter the horticultural value network during primary production in field or greenhouse via irrigation, at harvest, during processing and distribution or in the home kitchen/restaurant. The hurdle approach involves combining several mitigating approaches, each of which is insufficient on its own, to control or even eliminate pathogens in food products. Since the food chain system for leafy green vegetables contains no absolute kill step for pathogens, use of hurdles at critical points could enable control of pathogens that pose a human health risk. Hurdles should be combined so as to decrease the risk due to pathogenic microbes and also to improve microbial stability, shelf-life, nutritional properties and sensory quality of leafy vegetables. The hurdle toolbox includes different options, such as physical, physiochemical and microbial hurdles. The goal for leafy green vegetables is multi-target preservation through intelligently applied hurdles. This review describes hurdles that could be used for leafy green vegetables and their biological basis, and identifies prospective hurdles that need attention in future research.

## Introduction

Vegetables are an essential part of the human diet and an important source of minerals and vitamins. They are often eaten raw or only minimally processed (Goodburn and Wallace, 2013), particularly leafy green vegetables (LGV) such as baby leaf spinach and Swiss chard, rocket and different types of lettuce. Consumption of fresh produce in general has increased over the past two decades (Olaimat and Holley, 2012). At the same time, the number of foodborne disease outbreaks associated with consumption of LGV has increased (Castro-Ibáñez et al., 2017). The chief bacterial pathogens associated with these outbreaks are Shigatoxin-producing *Escherichia coli* (STEC), *Salmonella* spp., *Yersinia* spp. and *Listeria monocytogenes* (Tables 1, 2). In addition to being vehicles of human pathogens, ready-to-eat (RTE) salads may also be vehicles for bacteria with genes coding for resistance to specific antibiotics (Campos et al., 2013).

**Table 1:** Examples of foodborne disease outbreaks linked to leafy vegetables from 2000 onwards, starting with the most recent.

**Year**	**Country**	**Product/crop**	**Pathogen**	**Isolation from implicated food**	**Cases (deaths)**	**References**
2016	U.K.	Salad mix	*E. coli* O157	No	161 (2)	Public Health England (PHE), 2016
2015/2016	U.S.	Ready-to-eat salad mix	*L. monocytogenes*	Yes	19 (1)	CDC, 2016; Self et al., 2016
2015	U.K.	Pre-packed salad	*E. coli* O157	No	38	Public Health England (PHE), 2015
2014	Norway	Salad mix (probably radicchio rosso as it has a longer shelf-life than other ingredients in salad mix)	*Y. enterocolitica* O:9	No	133	MacDonald et al., 2016
2013/2014	Norway	RTE salad mix (imported rocket, baby spinach and red rhubarb, washed and bagged in Norway)	*Salmonella* Coeln (*Salmonella enterica* spp. *enterica*)	No	26	Vestrheim et al., 2016
2013	U.S.	Romaine lettuce (from Mexico)	*Cyclospora*	No	631	Buss et al., 2016a,b
2013	Sweden	Mixed green salad served at restaurant	*E. coli* O157	No	19	Edelstein et al., 2014
2012/2013	Canada	Lettuce served at fast food chains	*E. coli* O157	No	31	Tataryn et al., 2014
2012	Finland	Frisée salad from Netherlands	*Cryptosporidium parvum*	No	>250	Åberg et al., 2015
2012	U.K.	Pre-cut mixed salad (including leaves from growers in the UK, Spain, Italy and France)	*Cryptosporidium parvum*	No	>300	McKerr et al., 2015
2012	U.S.	Bagged salad (romaine, iceberg lettuce, cabbage, carrots)	*E. coli* O157	No	17 (2)	Marder et al., 2014
2012	U.S.	Pre-packed organic spinach and salad mix	*E. coli* O157	Yes	33	CDC, 2012b
2011	Norway	Salad mix containing radicchio rosso	*Y. enterocolitica* O:9	No	21	MacDonald et al., 2011
2011	U.S.	Romaine lettuce	*E. coli* O157	No	58	CDC, 2012a; Slayton et al., 2013
2010	U.S.	Romaine lettuce (shredded)	*E. coli* O145	Yes	27	CDC, 2010; Taylor et al., 2013
2010	Denmark	Lollo bionda lettuce (from France)	Entero-toxigenic *Escherichia coli*	Yes	260	Ethelberg et al., 2010
2008	Finland	Ready-to-eat iceberg lettuce imported from Central Europe	*Salmonella* Newport/Reading	No	107 (2)	Lienemann et al., 2011
2007	Sweden	Baby spinach (imported)	*Salmonella*. Paratyphi B variant Java (*Salmonella* Java)	No	172^a^	Denny et al., 2007
2006	U.S.	Spinach	*E. coli* O157	Yes	191 (5)	CDC, 2006; Grant et al., 2008
2005	Sweden	Iceberg lettuce	*E. coli* O157	No	135	Söderström et al., 2008
2005	Finland	Iceberg lettuce (from Spain)	*Salmonella Typhimurium* DT 104B	Yes	60	Takkinen et al., 2005
2005	U.K.	Iceberg lettuce	*Salmonella* Typhimurium DT104	No	96	HPA, 2005a,b
2004	U.K.	Lettuce	*Salmonella* Newport	No	>360	Gillespie, 2004; HPA, 2004; Irvine et al., 2009
2004	Denmark, Norway, Sweden	Rocket (from Italy)	*Salmonella* Thompson	Yes	100	Nygård et al., 2008
2003	U.K.	Iceberg lettuce (from Spain)	*Salmonella* Braenderup	Yes	29	Gajraj et al., 2012
2000	U.K.	Lettuce	*Salmonella* Typhimurium DT 104	No	361	Horby et al., 2003
2000	Iceland, U.K., Netherlands, Germany	Lettuce	*Salmonella* Typhimurium DT204b	No	392	Crook et al., 2003

a *Cases also in UK and Denmark*.

**Table 2:** Summary of characteristics of microorganisms of most concern on leafy greens from a food safety perspective.

**Microorganism**	**Size (**μ**m)**		**Motility**	**pH range**			**Temperature range (**^**°**^**C)**			**Metabolism**	**Energy source**	**Environment**	**References**
	**Diameter**	**Length**		**Min**.	**Optimum**	**Max**.	**Min**.	**Optimum**	**Max**.				
STEC	1.1–1.5	2.0–6.0	Peritrichous flagella or nonmotile	3.5		9.0	15	21–37	45	Facultative anaerobic	Glucose and other carbohydrates	Primary reservoir is the bovine intestinal tract	Park et al., 1999; Kaper et al., 2004; Brenner, 2005
*Salmonella*	0.7–1.5	2.0–5.0	Peritrichous flagella	4.0	6.6–8.2	9.0	5.3	37	45	Facultative anaerobic	Amino acids, nitrate, nitrite, ammonia	Warm- and cold-blooded animals, humans, eggs, milk and dairy products	Matches and Liston, 1968; Page and Solberg, 1980; Brenner, 2005; Jay et al., 2005
*Yersinia*	0.5–0.8	1.0–3.0	Peritrichous flagella; motile at temperatures below 30°C. *Y. pestis* always nonmotile	4.0	7.0–8.0	10.0	−2	28–29	45	Facultative anaerobic	Sucrose (cannot utilize rhamnose)	Domestic and wild mammals and birds feces, water, vacuum-packed meats, seafood, vegetables, milk, pigs (most prominent reservoir)	Bottone and Mollaret, 1977; Hanna et al., 1977; Stern et al., 1980; Brenner, 2005; Jay et al., 2005
*Listeria*	0.4–0.5	1.0–2.0	Peritrichous flagella, tumbling motility	4.3	6.0–8.0	9.4	>0	30–37	45	Facultative anaerobic	Require carbohydrate as primary energy source, glucose is preferred source	Soil, vegetation, meat (fresh and frozen), water, poultry, and cattle	Petran and Zottola, 1989; Pine et al., 1989; George et al., 1996; te Giffel and Zwietering, 1999; Jay et al., 2005; De Vos et al., 2009
*Shigella*	1.0–3.0	0.7–1.0	Nonmotile	5.0	6.0–8.0	9.0	10	37	48	Facultative anaerobic	Glucose and other carbohydrates	Intestinal tract of humans and primates	Small et al., 1994; Brenner, 2005; Jay et al., 2005
*Cryptosporidium*	4.0–6.0		Gliding motility	2.0		10.0	4		15 (they can survive at higher temperatures, but oocyst infectivity is inactivated at higher temperatures)	Obligate intracellular coccidian parasite	Amylopectin is the energy reserve needed for excystation and invasion of host cells	Surface waters. Sporulated oocysts are shed in the feces of infected hosts. By contamination of the environment, food or water, oocysts can be ingested by other suitable hosts	Vetterling and Doran, 1969; Fayer and Ungar, 1986; Rose et al., 1997; Brush et al., 1998; Smith and Rose, 1998; Jay et al., 2005; King et al., 2005; Wetzel et al., 2005

Ready-to-eat salads are a convenient way to ensure intake of vegetables, but consumers need to be confident that the products are safe to eat. However, analysis of RTE salads in Finland has shown that the bacterial quality and safety of packaged fresh LGV is often poor (Nousiainen et al., 2016). It is unclear whether production, processing or distribution practices (or all of these) are responsible for RTE salads of substandard quality, but there is clearly a need for improvements regarding suppression of pathogenic microbes within the entire horticultural value network for LGV, from farm to fork.

Ensuring the microbial safety of fresh LGV presents a unique challenge because the products are consumed raw, with no kill step of pathogens (e.g., heating) at any point in the chain to prevent transmission (Gil et al., 2015). Attempts has been done with aqueous ozone, that could potentially lead to a log 2 reduction of *E. coli*, without detrimental effects on the chemical characteristics of the vegetables (Karaca and Velioglu, 2014). In some countries disinfectant wash, e.g., a chlorine solution, is an option, but it is unclear if this is sufficient to guarantee the food safety due to the risk of internalized bacteria that cannot be reached. There are also indications that the bacteria can adapt to the sanitizer stress with increased resistance to hydrogen peroxide and calcium hypochlorite (Kyle et al., 2010) in combination with increased expression of virulence determinants—potentially increasing the risks. Furthermore, standard commercial washing and distribution conditions may be insufficient to reliably control human pathogens on fresh produce (Hutchison et al., 2017). Pathogens can be adept at adhering to leaf surfaces and potentially penetrating into internal leaf structures, which limits the usefulness of chemical sanitation methods in preventing pathogen transmission via contaminated produce (Lynch et al., 2009).

**Figure 1:**
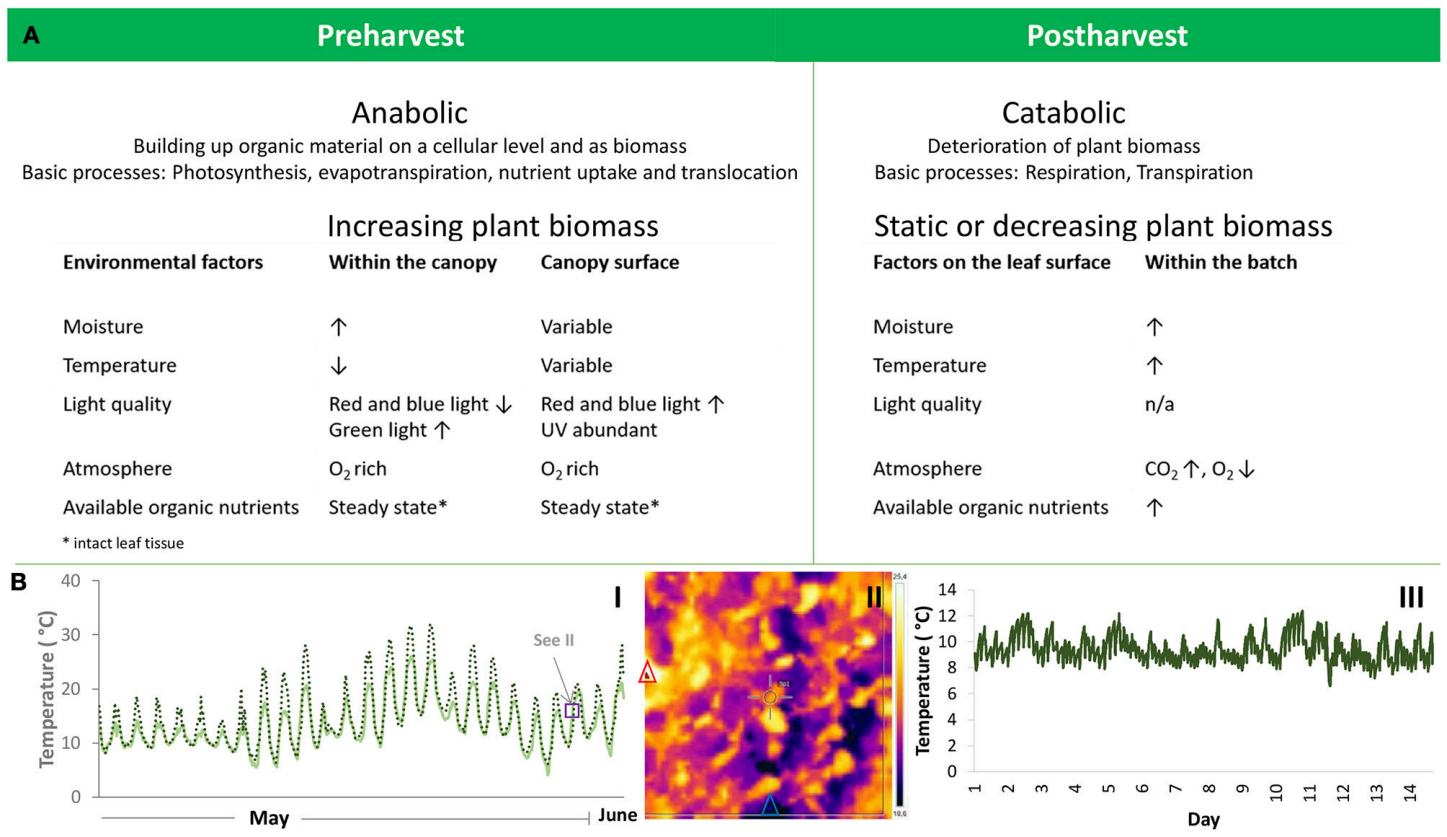
Environmental conditions prevailing during pre- and post-harvest of leafy vegetables and affecting the fate of phyllosphere organisms. **(A)** Change of the plant matrix from pre- to post-harvest and related alterations in the physio-chemical phyllosphere environment. **(B)** Temperature as an example of fluctuations in the phyllosphere environment during pre-harvest (I: field environment; II canopy surface) and post-harvest (III: household refrigerator). I: Diurnal changes in atmospheric temperature (solid line) and crop stand temperature near the soil surface (broken line) monitored in southern Sweden during 3 weeks in May and June 2014 in a baby leaf spinach crop. Atmospheric temperature was recorded once per hour and crop stand temperature every 30 min. The purple box indicates the time window for assessment of leaf temperature displayed in II. II: Leaf surface temperature of field-grown baby leaf spinach. The image was taken with an infrared camera. The red and blue triangles indicate the sites with the highest and lowest temperatures in the section. III: Temperature fluctuations in a household refrigerator where temperature was recorded every 5 min during a 14-day period. (Pictures and original data in the figures have previously not been published. Photo by Beatrix Alsanius).

Critical steps in the value network for LGV are: (1) Primary production, (2) harvest, (3) washing and processing, (4) packaging, and (5) handling, distribution, display, and retail. It is vital to take account of all these steps when considering microbial safety. For example, in an *E. coli* outbreak in Sweden in 2005 with 135 cases, production conditions were implicated in the safety of the final produce because cattle shedding STEC at a farm upstream from the irrigation point resulted in STEC-contaminated lettuce (Söderström et al., 2008). Irrespective of contamination source, there are numerous abiotic and biotic variables that influence conditions on the surface of leafy greens (Figure 1). This in turn modulates continued growth and survival of introduced pathogenic microbes (Tomás-Callejas et al., 2011). For example, *E. coli* and *Salmonella* achieve high numbers on young leaves, suggesting that leaf age affects pre-harvest and post-harvest colonization by pathogens (Brandl and Amundson, 2008).

Overall, microbial hazards are significant for fresh produce. Therefore, ways to reduce sources of contamination and a deeper understanding of pathogen survival and growth on fresh produce are required to reduce the risk to human health and the associated economic consequences (Alegbeleye et al., 2018). As there is no single solution for ensuring safety of LGV, the hurdle approach appears to be an appropriate treatment system for this market section of produce. In the next sections of this review, we provide essential background by describing disease outbreaks and then define the hurdle concept. In terms of scope, our review assesses abiotic and biotic parameters relevant for bacterial pathogens on leafy greens, in order to understand the biological underpinnings of hurdles, and summarizes existing hurdles. We also consider potential hurdles and the research needed to develop these.

## Nature of recorded disease outbreaks

EFSA (2013) ranked combinations of pathogens and foodstuffs of non-animal origin including fruit, vegetables including leafy green, salads, seeds, nuts, cereals, herbs, spices, fungi, and algae (EFSA, 2013). The food safety concerns are a consequence of that many foods of non-animal origin are consumed as ready-to-eat foods in which the constituents are raw or minimally processed (e.g., fresh-cut and prepacked).

EFSA noted that food of non-animal origin such as vegetables were implicated in 10% of the reported outbreaks during 2007–2011. However, those outbreaks were larger and more serious as they involved 26% of the cases, 35% of the hospitalizations, and 46% of the deaths. The huge VTEC O104 outbreak linked to consumption of contaminated fenugreek sprouted seeds in Germany 2011 as well as other European countries influenced these numbers (Beutin and Martin, 2012). However, if excluding this outbreak, still 18% of the cases were linked to consumption of foods of non-food origin.

The risk ranking was done based on following criteria: (a) Strength of association between consumption of the foodstuff and disease associated with the specific pathogen; (b) Incidence of illness and burden of the disease; (c) The dose response curve—high rank if the curve indicated that low doses could cause infection and disease; (d) Amount consumed of the vegetable; (e) Prevalence of contamination found on the vegetable, and (f) Growth potential of the pathogen during the shelf life.

Subsequently, the risk ranking identified *Salmonella* in leafy greens eaten raw, as the main concern. Moreover, the combinations of *Salmonella* spp. and bulb or stem vegetables; tomatoes; and melons was also a concern. The combination of pathogenic *Escherichia coli* and fresh legumes or grains were also suggested posing a risk.

A total of 29 outbreaks that occurred world-wide between 2000 and 2016 are listed in Table 1. Bacteria were responsible for most of these outbreaks, with STEC and *Salmonella* being the most commonly implicated infectious agents. The STEC outbreaks were more commonly reported in the U.S. and Canada, while the majority of the *Salmonella* outbreaks were reported in European countries (Table 1). Almost half of all cases were associated with consuming LGV outside the home, e.g., in restaurants, fast food outlets or school cafeterias. In general, vegetables are more frequently consumed by women (Takkinen et al., 2005; CDC, 2006, 2012b; Söderström et al., 2008; MacDonald et al., 2011; Marder et al., 2014) and this is reflected in the majority of cases reported being female (Table 1). Between 2000 and 2008, the crops associated with listed outbreaks were unspecified “lettuce,” iceberg lettuce, rocket and (baby) spinach, while in more recent epidemics salad mixes (“RTE salad mix,” “pre-cut mixed salad,” “bagged salad,” “pre-packed salad”) and romaine lettuce were implicated (Table 1).

Considering the ephemeral nature of leafy vegetables, which means that they need to be eaten quickly or disposed of, in conjunction with the fact that the incubation period of enteric pathogens can be several days, it is not surprising that the causative pathogen is only detected in suspected produce in less than one-third of cases (Table 1). This inability to conclusively link a causal agent to a disease outbreak is a hallmark of many foodborne illnesses. Therefore, most outbreak investigations rely on epidemiological evidence to obtain an indication of the possible contamination source and transmission path. In Finland, restaurants are advised to keep frozen samples of served foods for at least 2 weeks, to enable microbiological investigations after possible outbreaks (Åberg et al., 2015). This system for internal quality control should be encouraged in other countries and the storage period required should be extended to at least 4 weeks, since it typically takes more than 2 weeks from point of infection to confirmation (CDC, 2017). To further complicate source tracking, many LGV products implicated in outbreaks are mixes of varieties with different origins (MacDonald et al., 2011; McKerr et al., 2015). For example, in an outbreak of yersiniosis in Norway in 2011, the salad mix implicated contained four varieties of leafy vegetables originating from 12 suppliers in two countries, while the vegetables were mixed, washed, and packaged in Norway (MacDonald et al., 2011).

Identification of the offending produce enables subsequent trace-back investigations, which can identify potential suspects responsible for the original contamination further upstream within the value network (Horby et al., 2003; Slayton et al., 2013; Public Health England (PHE), 2015; Buss et al., 2016a,b). However, there have only been two outbreaks for which bacteria matching the outbreak strain have been isolated from environmental samples on, or in close proximity to, suspected farms (CDC, 2006; Grant et al., 2008; Söderström et al., 2008). Sweden and the U.S. experienced EHEC outbreaks in 2005 and 2006, respectively, and in both cases the outbreak strain was isolated from cattle (both countries) and feral swine (U.S. only) at or adjacent to the specific primary production site (CDC, 2006; Grant et al., 2008). Irrigation water contaminated with feces is often suggested to be the culprit for LGV contamination with pathogens (Ethelberg et al., 2010; Åberg et al., 2015). Furthermore, in a Finnish *Cryptosporidium* outbreak in 2012, it was speculated that contaminated water splash from heavy rain events during the growing period caused infection of growing produce in the field (Åberg et al., 2015).

The rapid distribution of foodstuffs via complex distribution networks has the capacity to distribute foodborne infection swiftly and widely. When only a few cases are infected by each pathogen in a country, outbreaks can be very difficult to detect locally or nationally. Therefore, international communication networks such as the Rapid Alert System for Food and Feed (RASFF) and genotyping approaches such as PulseNet are very important in outbreak investigations focusing on leafy vegetables distributed to different countries (Nygård et al., 2008).

## The hurdle concept

Food of animal origin can undergo a thermal process in order to inactivate human pathogens. Leafy green vegetables (LGVs) are eaten raw and cannot be treated the same way due to induced physiological damages and deterioration of the organoleptic properties. The limitation of disinfectants to completely eradicate food borne pathogens on fresh produce could partly be explained by the localization of bacteria in protected niches, cracks and crevices, or that they are even internalized and protected from direct contact (Sela and Manulis-Sasson, 2015). Secondly, biofilm formation on the plant's surface protects the pathogens from antimicrobial agents. The challenge is thus to design a network for safe LGV from field to fork that preserves freshness, prolongs shelf-life and maintains quality. However, there is no single risk mitigation measure that can achieve acceptable food safety for LGV. This lack of efficient kill-step means that the fresh produce industry must rely on preventive measures such as good agricultural practice (GAP) and hazard analysis of critical control points (HACCP) (Sela and Manulis-Sasson, 2015). This approach requires decisions based on evidence and a framework for describing the decision process within the supply chain (Monaghan et al., 2017).

Hurdles, or “the hurdle approach,” is a strategy employed in the food chain for meat and animal-based products (Leistner, 2000), but can be adopted as a novel approach for LGV. The hurdle approach has been proposed to some extent before. Some studies have suggested the use of natural agents as hurdles, other the use of bacteriocins, modified athmosphere and a strict management of temperature and storage times (Wadamori et al., 2017). But, regardless of the type of hurdles, the basis of the hurdle concept is a combined approach incorporating two or more practices (or hurdles), each of which may not be sufficient to control foodborne pathogens by itself, but together they can reduce or eliminate microbial hazards (Leistner, 2000). The main aim of the hurdle approach is to achieve microbial safety, product stability, organoleptic and nutritional quality, and economic viability of food production. “Fit for purpose” is key with the approach, as each aim must be achieved without detriment to the other aims.

The magnitude of microbial reduction achieved through use of hurdles will vary depending on circumstances in primary production, *e.g*. whether a large microbial burden (both pathogens and spoilage organisms) is assumed or not. For milk, an example of a foodstuff with high microbial burden, the key hurdles are pasteurization, cool storage and protection against cross-contamination. pasteurization of milk (72°C for 15 s) can reduce the bacterial load by around 7 log_10_ units for *L. monocytogenes, Salmonella Typhimurium*, and pathogenic *E. coli* (Pearce et al., 2012). However, pasteurization is not an option for fresh leafy greens. Instead, the equivalent key element of safe LGV production must be to minimize the pathogen and spoilage burden pre-harvest and to maintain a low burden throughout the whole food value network.

## Pathogenic organisms most likely to be found on LGV

Shigatoxigenic *E. coli, Salmonella, L. monocytogenes, Yersinia* spp., *Shigella*, and *Cryptosporidium* are the pathogens of greatest concern as regards LGV and food safety (Table 2). Apart from the protozoan *Cryptosporidium*, these are all facultative anaerobic bacteria and the majority are motile. They can grow across a wide pH range, from strongly acidic (pH 2 and 3.5 for *Cryptosporidium* and STEC, respectively) to alkaline (pH 9–10). They have an optimum temperature for growth of between 21 and 37°C, although some are capable of growth at much lower temperatures, close to 0°C (e.g., *Y. enterocolitica* and *L. monocytogenes*) (Table 2). Some are widespread in the environment (i.e., *L. monocytogenes*) and most are found in the gut and feces of warm-blooded animals.

## Abiotic factors to be considered in the hurdle approach

### Temperature

Environmental conditions prevailing during pre- and post-harvest of leafy vegetables and affecting the fate of phyllosphere organisms are summarized in Figure 1. One of the most influential drivers of plant and microbial activities and growth is temperature. Within the path taken by LGV through the food system, there are three marked phases that can be delineated, namely pre-harvest, harvest, and post-harvest. Each stage displays distinct temperature kinetics due to the physiological status of the plant material and the surrounding ambient temperature conditions.

For a human pathogenic bacterium residing in the phyllosphere, the most critical factor is the temperature on the leaf surface, which is a function of wind speed, air temperature, transpiration rate, and diffusion resistance in plant tissues (Gates, 1968; Larcher, 2003).

There are complex interactions between the parameters influencing leaf temperature. For example, cushion, and rosette plants (e.g., baby spinach, Swiss chard, rocket) form a closed canopy close to the soil surface and are less prone to convection energy losses (cooling by wind), which in turn allows temperatures to rise above air temperature (Figure 1). However, this effect only applies to the interior of the canopy, while leaves in the exterior canopy border interfacing with the atmosphere are more exposed to convection losses (Larcher, 2003). Temperature relationships relating to plant architecture have not been studied specifically for head-forming leafy vegetables, such as different types of lettuce.

Horticultural production systems incorporate many management practices, including mulching, row covering and low tunnels, which significantly alter the thermal environment of cropped plants (Krug et al., 2002). For example, temperature fluctuations (amplitude) are greater under black polyethylene film and lower under white polyvinylchloride film (Takakura and Feng, 2002). Apart from the thermal impact, mulch using polymeric films shields the crop from the soil surface and thereby reduces contamination of the foliage through soil splash.

Harvesting is a decisive intervention in the physiological and thermal conditions of leafy vegetables. From a physiological perspective, harvesting involves detachment of the leaf and hence interruption of the water flow continuum. Harvested leaves are subject to respiration, leading to a temperature increase, and to dehydration, especially if the relative humidity of the surrounding air is low. To maintain organoleptic properties, leaf integrity and the bioactive compound content, harvested leaf biomass is generally stored under cool conditions during transport, processing and packaging, distribution, retail and before preparation and consumption. Leafy green vegetables are highly perishable and any signs of decay strongly diminish their appeal to consumers. Moreover, during decay they also lose organic nutrients from the leaf matrix, favoring microbial growth. Harvested leafy vegetables should thus be cooled as soon as possible to prevent water loss and respiration and to deter microbial proliferation (Paull, 1999). Different cooling methods are available, e.g., room cooling, forced air cooling, hydro cooling, vacuum cooling, water-spray vacuum cooling etc. (Thompson et al., 2002). These methods differ in terms of their cooling efficiency, and thus in their effect on the final produce quality, so the method employed should be chosen with respect to target produce, infrastructure and economic considerations at the processing facility. In addition, positioning of harvested trays affects the rate of cooling, which progresses more rapidly in trays placed in the middle of a stack than in trays at the top or bottom of the stack (Rediers et al., 2009).

There is of course a time lapse between harvesting and the onset of adequate cooling temperatures. In this context, seasonality is important. Onset of target cooling temperatures is delayed when harvest takes place at elevated outdoor temperatures, which can ultimately reduce produce quality and increase microbial proliferation (Rediers et al., 2009; Garrido et al., 2015). High outdoor temperatures during harvesting also promote respiration, so pre-cooling using a hydro or vacuum method may be preferable as this increases leaf water content (Garrido et al., 2015). In summary, important hurdles to consider are preferentially harvesting LGV at lower outdoor temperatures and using a pre-cooling method by which target temperature can be reached faster or respiratory water loss can be mitigated.

Post-harvest temperature management is the most critical hurdle for LGV. According to the University of Florida's Institute of Food and Agricultural Sciences (UF-IFAS) recommendations for processing, post-harvest temperature for LGV should not exceed 5°C (Garrido et al., 2004). In reality, however, there are large variations in the ability to maintain this target temperature throughout storage, processing, distribution, and retail of LGV.

Post-harvest temperature management can change in different phases. A comprehensive study by Rediers et al. (2009) found that temperature was well managed during the post-harvest path of endive until distribution to restaurants, whereupon breakdowns in temperature maintenance occurred, with temperature increases of up to 4°C. Considerable temperature fluctuations were also noted during up to 4 days of storage at restaurants (Rediers et al., 2009). In contrast, Zeng et al. (2014) found that low temperatures are often not maintained during transport, retail storage, and even display (Zeng et al., 2014). Transfer points within post-harvest paths seem especially vulnerable to failures to maintain low target temperatures (Koseki and Isobe, 2005).

There is strong consumer awareness that LGV need to be refrigerated (Jacxsens et al., 2015), but measurements of the actual temperature achieved during domestic storage (refrigeration) show that home refrigerators may be unable to maintain isothermal conditions consistently below the target 5°C (Figure 1). The type and design of the refrigerator, its placement within the kitchen (close to a heat source), operating conditions, the temperature setting and the number of door openings affect the temperature distribution within refrigerators (Laguerre et al., 2002). Despite the fact that LGV are highly perishable, appropriate domestic storage is not always achieved. A study by Marklinder et al. (2004) found that almost one-third of respondents stored RTE salads in the warmest locations of their refrigerator, where mean storage temperature was 6.2°C and the highest refrigeration temperatures recorded were 11.3–18.2°C (Marklinder et al., 2004). From a food safety perspective, such high refrigeration temperatures should be considered an abuse temperature regime under which many human pathogenic bacteria are capable of growth. Similar data have been reported previously (Carrasco et al., 2007), and in a recent study the risk of waste of fresh-cut iceberg salad (partly due to microbial aspects) increased manifold with higher fridge temperatures (Manzocco et al., 2017).

Few studies have examined the temperature dynamics of perishable produce at the point of display. Thomas et al. (2014) monitored temperature dynamics during 3 days at 24 schools using cooling wells, cooling plates or healthy carts. They found that the temperature of the LGV displayed varied substantially and that it remained below 5°C only in a “holding cooler.” The mean time above 5°C for the other devices they tested followed the order: loose cooling pan>cooling plate>cooling well>display cooler (Thomas et al., 2014).

The fate of foodborne pathogens inoculated onto different horticultural commodities eaten raw and exposed to different temperature regimes has been well analyzed. The majority of these studies have analyzed *E. coli* O157:H7, *Salmonella* and *L. monocytogenes* but the temperature regimes employed differ, although most have been conducted under isothermal conditions. It is important to note that the term “abuse” temperature' used in the literature does not consider one specific threshold, but is arbitrarily assigned in different studies. In general, the literature shows that proliferation of *E. coli* O157:H7 is slowed down at lower temperatures (8°C compared with 12°C), but that this decline can be negated by extended storage time (Luo et al., 2009). This pathogen does not grow, but survives, at 5°C (Luo et al., 2010). Similar results have been reported for *Salmonella* Hadar inoculated on washed and shredded white cabbage, with survival but no growth at 4°C, but growth observed at 12°C and 20 °C (Piagentini et al., 1997; Delbeke et al., 2015).

In a study where *E. coli* O157:H7 gfp+, *L. monocytogenes*, and *Y. enterocolitica* were inoculated onto baby spinach and stored at 8 and 15°C for 7 days, only populations of *Y. enterocolitica* increased significantly and exclusively under temperature abuse (15°C) (Söderqvist et al., 2017). Intactness of LGV has an impact on nutrient availability, and hence proliferation of contaminants. Khalil and Frank (2010) found that the occurrence of *E. coli* O157:H7 was particularly promoted on bruised parsley and bruised spinach at 12°C during 4 days of storage and that growth of *E. coli* O157:H7 on bruised spinach, but not on bruised coriander cilantro, romaine lettuce and parsley, was selectively promoted after 3 days of storage at 8°C (Khalil and Frank, 2010). Interestingly, processing treatment affects the fate of *E. coli* O157:H7 during storage at different temperature regimes (Lopez-Velasco et al., 2010). At low temperature (4°C), *E. coli* O157:H7 is internalized into cut iceberg lettuce tissue more efficiently than at higher temperatures (7, 25, and 37°C) (Takeuchi and Frank, 2000).

In a study that simulated temperature conditions during transport and retail, *E. coli* O157:H7 counts were found to be approximately 3 log colony-forming units (CFU)/g in transport and the population increased by 0.1 to 3.0 log CFU/g during retail storage, but did not increase further during retail display (Zeng et al., 2014). In similar temperature simulations, *L. monocytogenes* substantially increased under retail storage temperatures (by 3.0 log CFU/g) and retail display temperatures (by 1.1 log CFU/g) (Zeng et al., 2014). Considering the interaction between human pathogens and temperature conditions, a hurdle approach for LGV must include strict temperature management to avoid temperatures that can support growth of pathogens. Access to data on the temperature history of LGV batches can be an important complement, so that storage times can be adjusted according to previous temperatures experienced by the produce.

### Irradiation

Light is a critical factor in crop production, due to its impact on plant biology. Light quality (spectral distribution) and quantity (day light integral, light intensity) and diurnal changes (light period/day length, sunfleck and diurnal artificial light modulation) are all important in governing plant physiological and metabolic processes. By extension, parameters that influence vital plant processes will also have an indirect impact on phyllosphere microbiota residing on the leaf surface. This indirect effect is primarily mediated by nutrients leached from the leaf onto the surface, but temperature and humidity are secondary light effects to be considered, as they have a direct impact on bacteria (Alsanius et al., 2017). Interestingly, recent findings demonstrate that light can have a direct influence on the metabolism of phyllosphere bacteria, even those that are nonphototrophic (Gharaie et al., 2017).

Only a few studies have considered the impact of light source, quantity and quality on the epiphytic phyllosphere microbiota. Of these, most consider light quality interactions, while light source and quantity receive less attention. It has been shown that variations in cumulative photosynthetically active radiation (PAR) in a lettuce crop under field conditions cause a shift in the abundance of bacterial families, with e.g., Betaproteobacteria decreasing and Gammaproteobacteria increasing between the highest and lowest light regimes (Truchado et al., 2017). However, it is not clear whether this effect is due to variations in PAR, light quality or other abiotic factors associated with shielding. Light quality studies have predominantly focused on the impact of different UV-bands on epiphytic bacteria. An impact of light source and light quality has been demonstrated in greenhouse experiments, where UV-B irradiation changed the microbial phyllosphere community structure of various crops (Newsham et al., 1997; Jacobs and Sundin, 2001; Kadivar and Stapelton, 2003). However, pigmented phyllosphere bacteria are equipped with UV-B protection strategies, to mitigate DNA damage (Jacobs et al., 2003). Other protection strategies for preventing detrimental UV-A effects are based on quenching reactive oxygen species, e.g., using an avobenzone-like compound that absorbs UV-A found in *Methylobacterium*, a phyllosphere resident (Yoshida et al., 2017). Furthermore, blue light receptor domains have been found in several phyllosphere bacteria and human pathogens, which indicates that the ability to detect blue light may be more common than previously thought (van der Horst et al., 2007; Ondrusch and Kreft, 2011; Wu et al., 2013; Río-Álvarez et al., 2014; Alsanius et al., 2017; Gharaie et al., 2017). Recent results indicate that both light quality and nutritional factors are critical for bacterial nutrient utilization and propagation of phyllosphere bacteria and may also dictate colonization success (Gharaie et al., 2017).

In the context of light microbe interactions in crop stands, it is worth mentioning that the ambient light quality conditions only reflect those to which the top of the crop is exposed. When a covering canopy has developed, this results in light quality stratification as basal leaves are shaded (Alsanius et al., 2017). While managing irradiation to mitigate enteric pathogens may not be feasible in a field setting, it may have some potential in a greenhouse setting where light conditions are more controlled. However, much work still needs to be done in order to determine whether light manipulation is an achievable hurdle and irradiation currently does not make any practical contribution as a hurdle.

### Water availability and moisture/humidity

Leaf moisture is a function of the dynamics of air and leaf temperature and relative air humidity, and thus also of crop transpiration. Leaf wetness, by contrast, is very difficult to define, because various portions of leaves and canopies are wet and dry at different times (Huber and Gillespie, 1992). Leaf moisture is also dependent on plant morphological properties, such as leaf topography, cuticle morphology and composition, and the density of trichomes. The fate of an bacterium on the leaf surface is dependent on the leaf surface structure in combination with the amount of nutrients and water available (Monier and Lindow, 2005). If bacterial cells arrive on the leaf surface within droplets, such as rain splash or irrigation water droplets, they can move with these droplets across the leaf surface and aggregate at sites where water remains for the longest time during subsequent drying (Monier and Lindow, 2005). The crop surface is exposed to sunlight and desiccation, which leads to shorter survival times of pathogenic microorganisms on crops compared with in water and soil. If pathogenic microorganisms remain viable in the soil, they are able to re-contaminate plants during irrigation and rainfall (Steele and Odumeru, 2004). Although there is evidence that both *S. enterica* and *E. coli* O157 have the ability to colonize some plant species, they usually fail to grow on leaves under dry conditions (Lindow and Brandl, 2003).

One prominent cultural management measure affecting leaf moisture and relative humidity in the crop stand is irrigation. Moreover, irrigation water of inferior hygienic quality (surface water, reclaimed water, non-treated and treated sewage water) is listed as one of the most decisive vehicles for human pathogen transmission to LGV. As leafy vegetables have a shallow root system, they need to be irrigated regularly (e.g., every 3 days under sunny and warm Scandinavian conditions, with an irrigation water volume of 5 mm/event). Bacterial pathogens contained in surface water used for irrigation are generally re-isolated from the surface of the irrigated crop (Islam et al., 2004; Ijabadeniyi et al., 2011). Irrigation water may also mediate internalization of human pathogens into the leaf tissue, using the stomata as points of entry (Kroupitski et al., 2009a). Several measures have been suggested to prevent transmission of human pathogens through irrigation water (Allende and Monaghan, 2015). First, it is important to monitor pathogen and indicator species in water sources. Second, available physical and chemical water treatment methods to remove human pathogens from the irrigation water source should be applied. Third, the irrigation method used should be considered (Allende and Monaghan, 2015). Methods such as subsurface drip irrigation avoid direct contact with the edible parts of the crop, but more knowledge is required on the risks posed by root contamination and internalization of bacteria. Soil splash created by “rain-sized” water droplets can transfer enteric bacteria from soil to leaves (Monaghan and Hutchison, 2012), but drip and overhead sprinkler irrigation do not influence the survival of *E. coli* O157:H7 on the lettuce leaf surface (Moyne et al., 2011). This means that there is a complex web of conditions affecting the microbial quality of irrigation water (Pachepsky et al., 2011; Liu et al., 2013).

### Nutrients

Phyllosphere bacteria, including introduced pathogenic bacteria, must have access to nutrients in order to either sustain or grow populations. There is a lack of published data regarding nutrients encountered on the surface of LGV, but well over 180 plants have been assessed to establish the kinds of nutrients that may be present in the phyllosphere (Tukey, 1970). Therefore, predictions concerning the nutritional environment of LGV surfaces can be made based on generalities established for the wide variety of plants already examined (Morgan and Tukey, 1964; Tukey, 1966, 1970). The types of nutrients that are commonly present on the leaf surface can be categorized into inorganic and several classes of organic molecules (Morgan and Tukey, 1964). Inorganic elements or ions include boron, calcium, copper, iron, sodium, magnesium, nitrogen, phosphorus, silica, strontium, sulfur, and zinc (Tukey and Mecklenburg, 1964; Mecklenburg et al., 1966; Tukey, 1966, 1970).

Organic molecules include most of the known amino acids (Tukey, 1966, 1970; Rodger and Blakeman, 1984), as well as many organic acids, with a preponderance of tricarboxylic acid (TCA) cycle intermediates such as citric, fumaric and succinic acid (Morgan and Tukey, 1964; Tukey, 1966, 1970). Carbohydrates are of especial interest, due to their ability to readily support growth of enteric bacteria such as *E. coli*, and dominant phyllosphere sugars are fructose, glucose and sucrose (Morgan and Tukey, 1964; Tukey, 1966, 1970; Fiala et al., 1990; Dik et al., 1992; Mercier and Lindow, 2000; Leveau and Lindow, 2001; Aruscavage et al., 2010). Phylloplane nutrients may be of endogenous origin (Brown, 1922; Morgan and Tukey, 1964; Tukey and Mecklenburg, 1964; Mecklenburg et al., 1966; Leveau and Lindow, 2001), through passive leaching from within leaves themselves, or of exogenous origin, via deposits such as pollen, insect frass, and aphid honeydew (Rodger and Blakeman, 1984; Dik et al., 1991, 1992; Stadler and Müller, 2000). Two distinct hallmarks of the phyllosphere with respect to nutrients are nutrient scarcity and heterogeneous distribution, which represents a challenge for phyllosphere bacteria (Frossard et al., 1983; Haller and Stolp, 1985; Fiala et al., 1990; Leveau and Lindow, 2001). The heterogeneous distribution of nutrients indicates that nutrients leach in a non-random fashion across the leaf surface, with cracks and natural openings representing sites where greater amounts of nutrients are present (Leveau and Lindow, 2001). There is direct evidence that these phyllosphere nutrients are taken up and utilized by microbial residents and also that they represent an important source supporting microbial activities (Frossard et al., 1983; Rodger and Blakeman, 1984; Leveau and Lindow, 2001). Activity of fungi and bacteria are often restricted on the leaf surface due to nutrient limitations and there is a positive correlation between the amount of available nutrients and microbial growth (Bashi and Fokkema, 1977; Fokkema et al., 1979; Rodger and Blakeman, 1984; Mercier and Lindow, 2000). Leaching is enhanced by high water content and a moist or wet leaf releases more nutrients into the phyllosphere than a drier counterpart (Tukey, 1966). Wounds and injuries that breach the leaf surface, whether by biological or mechanical means, increase nutrient availability, with a concomitant increase in growth of microbes on the plant surface, including *E. coli* (Tukey and Morgan, 1963; Dingman, 2000; Jablasone et al., 2005; Aruscavage et al., 2008, 2010). Consequently, processing practices that inflict wounds or injuries, such as minimal processing of leafy greens in the form of chopping and shredding, cause a nutritional pulse that increases growth of *E. coli* and other resident microbes (Brandl, 2008). In conclusion, approaches to mitigating the risk of foodborne illnesses associated with produce and produce spoilage should include practices that aim to decrease leaching of nutrients. Considering that leaching is greatest from leaves that are injured, older and/or wet (Morgan and Tukey, 1964; Tukey, 1970; Aruscavage et al., 2010), then appropriate hurdles for enteric bacteria would be to avoid extended post-harvest storage periods and to avoid post- and processing approaches where leaves are either wetted for extended periods or subjected to practices that cause wounding or injuries.

### Oxygen/package atmosphere

Once harvested, leaves will continue to respire and if placed in sealed packages they will modify their own atmosphere due to consumption of ambient oxygen (O_2_), while releasing carbon dioxide (CO_2_). The aim of modified atmosphere packaging (MAP) is to extend the shelf-life of produce by slowing down respiration of the leaves. This is achieved by altering the gaseous environment, which can be accomplished either by harnessing innate produce respiration (passive MAP) or by adding or removing gases to manipulate the levels of O_2_ and CO_2_ (Oliveira et al., 2010). In general, a lower level of O_2_ (3–6%) and a higher level of CO_2_ (2–10%) induce a lower respiration rate in produce. Nitrogen may be used as a filler gas, to prevent collapse of the package (Sandhya, 2010). Of the gases used in MAP, CO_2_ is the only one with demonstrated antimicrobial activity olive (Oliveira et al., 2015). However, this inhibitory effect is not general and depends on many factors related to the microorganism, storage conditions, and product characteristics. Produce for which MAP is used may be vulnerable from a food safety perspective, as the associated extension of shelf-life may allow growth of pathogenic bacteria, while also inhibiting growth of organisms that usually make the consumer aware of spoilage by off-odors (Farber et al., 2003). The O_2_ and CO_2_ levels that develop inside the package due to different types of packaging films do not appear to affect survival and growth of E. coli O157:H7 on different types of fresh produce at 5°C, because growth of E. coli O157:H7 is predominantly dependent on temperature and to a lesser extent on atmospheric conditions (Abdul-Raouf et al., 1993; Oliveira et al., 2010; Abadias et al., 2012). Similar findings have been reported for *S. Typhimurium* and *S. choleraesuis* (Oliveira et al., 2010; Lee et al., 2011). The above also holds true for the psychrotrophic pathogen *L. monocytogenes*, which is of particular interest as it can grow at refrigerator temperatures. There is convincing evidence that *L. monocytogenes* populations grow unaffected, or persist, in MAP with high levels of CO_2_ at between 3 and 14°C (Kallander et al., 1991; Carlin et al., 1996; Jacxsens et al., 1999; Scifo et al., 2009; O'Beirne et al., 2015). Furthermore, high CO_2_ has been shown to promote growth of *L. innocua*, a bacterium which is often used as a valid model organism instead of the pathogenic *L. monocytogenes* (Geysen et al., 2006; Escalona et al., 2007). Thus, there is a risk that the level of *L. monocytogenes* will exceed the EC limit (100 CFU/g) if it is present even at low initial levels when stored in MAP. However, there are conflicting results, e.g., in one study an *E. coli* O157:H7 population was noticeably reduced in a high-oxygen (70 kPa O_2_) modified atmosphere (MA) compared with a low-oxygen (5 kPa O_2_) MA (Lee et al., 2011). It has also been shown that near-ambient air conditions support lower populations of *E. coli* O157:H7 when stored at 4°C than when stored in active MAP conditions with high CO_2_ (Sharma et al., 2011).

Apart from the proven importance of proper storage temperatures for fresh produce stored in MAP, other biological characteristics are also important. It has been shown that MAP increases the ability of different isolates of *E. coli* to survive gastric acid challenge on lettuce stored at abuse temperatures (≥15°C) (Chua et al., 2008). Storage under near-ambient air atmospheric conditions can potentially promote higher expression levels of *E. coli* O157:H7 virulence factors on lettuce, which could therefore affect the severity of *E. coli* O157:H7 infections associated with leafy greens (Sharma et al., 2011).

To summarize the role of atmosphere, it is likely that pathogens are more influenced by type of vegetable than by type of atmosphere (Jacxsens et al., 1999). If MAP is used to increase shelf-life, it should be applied with other preservation techniques to ensure inhibition of pathogens, as the MAP technique itself does not appear to represent an impediment to enteric bacteria (Scifo et al., 2009).

### pH

Human pathogenic bacteria have a narrow span of pH conditions in which they can survive on the leaf surface (Table 2). A pH value below 4.6 will inhibit growth and toxin production of many pathogens, however, many microorganisms, e.g., *Cryptosporidium* and STEC, are capable of growth or survival at pH values below this limit (Table 2). Lowering the pH of LGV to prevent growth of pathogenic bacteria may be a problem if it impairs taste due to fermentation (Leistner, 2000). Another issue is that some bacteria can develop resistance against acidity or can increase their virulence when exposed to environmental stress factors, such as osmotic stress, starvation, and suboptimal pH (Kroll and Patchet, 1992; Leistner, 2000). *Listeria monocytogenes* can develop resistance against acidity (pH 3.5) depending on other environmental conditions (Koutsoumanis et al., 2003). It has also been shown that *E. coli* O157:H7 can survive acidic conditions, with survival rates influenced by temperature (Conner and Kotrola, 1995). However, Menz et al. (2011) found that growth and survival of *E. coli* O157:H7 and *S. Typhimurium* in beer was prevented when the pH was lowered to 4.0 (Menz et al., 2011). In a study where *S. enterica* was spot-contaminated on lettuce leaves and lettuce pieces were then submerged in acid solution (pH 3.0), increased acid tolerance of attached bacteria compared with planktonic cells was observed (Kroupitski et al., 2009b). This means that altering the pH is probably not a viable hurdle in practical use to prevent growth of pathogenic bacteria on leafy vegetables at any step of the food chain.

### Biotic factors critical for the hurdle approach

In addition to abiotic factors such as temperature, nutrients, water and pH, biotic factors must be taken into consideration when trying to combine different hurdle approaches. In this review, we focus solely on the presence of other bacteria on the leaf surface.

### Microbiome associated with leafy greens

The native microbiota of leaves consists of millions of phylloepiphytes (bacteria of the phyllosphere; Lopez-Velasco et al., 2013). This diverse bacterial community varies due to morphological and chemical differences between plant genera. For example, Hunter et al. (2010) found that on all lettuce (*Lactuca sativa*) accessions they analyzed, the dominant species were from the Pseudomonadaceae and Enterobacteriaceae families, but that *Erwinia* and *Enterobacter* genera differed significantly between the accessions. Jackson et al. (2013) found that the amount of total culturable bacteria on salad vegetables ranged from 8 × 10^3^ to 5.5 × 108 CFU/g, with the cultured isolates belonging mainly to *Pseudomonas, Chryseobacterium, Pantoea*, and *Flavobacterium*. Culture-independent analysis of the same samples revealed that Gammaproteobacteria, Betaproteobacteria, and Bacteroidetes were the dominant lineages, including genera hosting plant pathogens such as *Pseudomonas, Ralstonia, Stenotrophomonas, Erwinia* and *Xanthomonas*, and genera hosting human pathogens such as *Serratia, Enterobacter, Bacillus* and *Staphylococcus*. There was no difference in microbial composition between organically and conventionally grown produce (Jackson et al., 2013).

Leafy green vegetables are significant vectors of human pathogens (Table 1). Pathogens that exist as endophytes are often involved in food-borne disease outbreaks of bagged lettuce and spinach, as they are more protected against external procedures such as washing (Uhlig et al., 2017). Although human pathogens are not adapted for growth in the phyllosphere, it has been shown that *E. coli* O157:H7 and *Salmonella* can survive at low levels over extended periods of time on plants in the field (Williams and Marco, 2014). Williams and Marco (2014) characterized bacteria on romaine lettuce grown in the laboratory and under field conditions and found that field-grown plants contained 10- to 100-fold higher bacterial load than laboratory-grown plants. Furthermore, field-grown plants contained significantly higher proportions of Gammaproteobacteria, represented primarily by Enterobacteriaceae and Moraxellaceae, among which the family Enterobacteriaceae in particular includes several well-known pathogens such as *E. coli* and *Salmonella*. In contrast, laboratory-grown plants were enriched with Betaproteobacteria, represented by the Comamonadaceae and Burkholderiaceae families (Williams and Marco, 2014). The relative quantities of *Erwinia, Acinetobacter* and *Alkanindiges* bacteria were significantly higher on field grown plants, whereas laboratory-grown plants carried significantly more representatives of *Comamonas, Limnobacter* and *Pelomonas* (Williams and Marco, 2014). It has been found that the uptake of *E. coli* O157:H7 can be mediated by the microbe itself. In a study by Solomon and Matthews (2005) in which lettuce plants were irrigated with *E. coli* O157:H7 or FluoSpheres, particles similar in size to bacterial cells but devoid of bacterial surface moieties, appendages or adaptive responses, it was found that both *E. coli* O157:H7 and FluoSpheres were internalized into growing plants and were present within root and leaf stem tissue. These findings suggest transport of spheres from the root up into edible tissue and, because the level of uptake of FluoSpheres and *E. coli* O157:H7 was similar, they indicate that entry of *E. coli* O157:H7 into lettuce plants may be a passive event (Solomon and Matthews, 2005).

### Antagonism and pathogens on leafy greens

Co-existence in bacterial communities is controlled by access to space, nutrient use and availability, production of antimicrobial compounds and other strategies to acquire resources. An invading microorganism, including a human pathogen, must successfully co-exist and compete with an already adapted and established bacterial community in order to establish (Lopez-Velasco et al., 2013). Biodiversity seems to affect this delicate equilibrium and a more diverse community is correlated with reduced levels of *Salmonella* colonization, presumably due to increased presence of antagonistic bacteria (Jackson et al., 2013). Plant microbiome transplantations using freshly transferred and cryopreserved field microbiota have been found to increase bacterial diversity and to result in a bacterial composition similar to field plant microbiota that seems to be stable over time (Williams and Marco, 2014). To examine the effect of an exogenous potential pathogenic organism on the indigenous bacterial communities in the phyllosphere, Williams and Marco (2014) inoculated *E. coli* O157:H7 onto laboratory-grown romaine lettuce plants containing or lacking the field plant microbiota and found that it resulted in significant shifts in the abundance of certain taxa in both groups. Specifically, bacterial species of the genus *Microbacterium* were significantly enriched on *E. coli* O157:H7-containing plants with transplanted phyllosphere microbiota. This genus has previously only been found on field-grown lettuce and has been shown to exhibit antagonistic activity against *E. coli* O157:H7, so it has been investigated as a biocontrol agent against fungal plant pathogens (Barnett et al., 2006; Pereira et al., 2009; Lopez-Velasco et al., 2012).

Lopez-Velasco et al. (2013) found that 15 genera found on spinach could reduce the growth rate of *E. coli* O157:H7 *in vitro*. The majority (83%) of these antagonistic bacteria belonged to the same taxonomic class as *E. coli* (Gammaproteobacteria), and the remainder belonged to Firmicutes (7%), Bacterioidetes (5%), Actinobacteria (2%), and a small proportion of Alphaproteobacteria and Betaproteobacteria. The mechanisms responsible for the antagonistic effect were reported to be acid production or nutrient competition, mainly of carbon sources. However, the reducing effect was significantly smaller when the antagonists were co-cultured with the pathogen on detached spinach leaves than when grown *in vitro* (Lopez-Velasco et al., 2013). *Escherichia coli* growth-stimulating bacteria belonging to Actinobacteria (33%), Bacterioidetes (33%), Alphaproteobacteria (26%), and Betaproteobacteria (6%) were also found. Research on microbe-human pathogen interactions in the phyllosphere is still in the fledgling stages, but insights gained from microbial control of plant pathogenic organisms indicate that such interactions are extremely intricate and complex. A holistic approach, including plant physiological factors and phenology, is therefore of great importance for successful hurdle development based on antagonistic mechanisms.

### Bacteriophages in food safety of leafy greens

During the last 10 years the possible use of bacteriophages has received more attention from the European Union food safety authorities as a practical food safety risk mitigation tool. One important question for regulatory agencies is whether the use of bacteriophages are acting as processing aids or food additives. Processing aids would be the case if reducing numbers of pathogens, but not preventing reinfection in the next steps of the food chain, while if considering use of bacteriophages as a food additive the foods treated with bacteriophages should be safe even in the case of recontamination. The European Food Safety Authority (Anonymous, 2009) issued an opinion on the use of bacteriophages and their modes of action. It appears to be not sufficient to conclude whether bacteriophages in general should be seen as a processing aid or food additive. It was recommended whether the use of bacteriophages should be considered a processing aid or food additive ought to be decided on a case by case basis for each food-pathogen-bacteriophage matrix.

Bacteriophages have two principal mechanisms for reducing number of food borne pathogens either by being temperate by causing “lysis of bacteria from within” where the lysis is caused by multiplication of phage within the bacteria i.e., having a temperate cycle or virulent where the phages are causing lysis without replication i.e., “lysis from without.” One attractive feature given the host specificity of bacteriophages is the ability to eliminate the targeted pathogens specifically while leaving the microflora unharmed (Moye et al., 2018). This in contrast to decontamination methods such as heat treatment or treatment with chlorine or organic acids that kill microorganisms indiscriminately and consequently with an impact on the quality and shelf life of the foodstuff. Kazi and Annapure (2016) and references therein noted that bacteriophages intended for use in food should be strongly lytic, their host range should cover all epidemiologically important strains of the target microorganism and be stable in the intended environment of use (Kazi and Annapure, 2016). If needed a mixture of phages should be used (phage cocktails) to ensure reduction, or preferably elimination, of relevant pathogens. Ensuring a longer shelf life biopreservation and biosanitation by removal of biofilms are further possible areas where bacteriophages could be useful.

Bacteriophages could be used both at the pre-harvest and harvest stages. For example, cattle might be treated with bacteriophages to reduce the shedding of *E. coli* O157: H7 (Sheng et al., 2006), according to this study the numbers were reduced but not eliminated. Hence, one could use the possibility of phage therapy for cattle grazing upstream in irrigation water catchment areas or grazing nearby–i.e., an indirect hurdle.

For vegetables, Sharma et al. (2009) found that bacteriophages reduced the number of *E coli* on lettuce and cantaloupe after 2 days of spraying with phage cocktail (ECP-100) (Sharma et al., 2009). It has been concluded that bacteriophages could reduce the number of pathogens by at least one log or 90% (Zaczek et al., 2015). So, at least for Enterohaemorhagic *E. coli, Salmonella* Sp, and *Listeria monocytogenes*, the use of bacteriophages could be useful as one amongst several hurdles to ensure food safety. The metabolic activity of the bacteria is very important for the efficacy of bacteriophages. For example, one should not expect a high effect if the temperature is low.

## Potential hurdles

Minimally processed foods are in increasing consumer demand possibly amplifying the consumer risks (Ohlsson and Bengtsson, 2002). In particular, risk mitigation and preservation options are needed to prolong shelf lives and to improve food safety for the following food groups; (i) minimally processed, convenient foods, (ii) chilled foods with “invisible technology”, (iii) healthful foods with less salt and/or fat, (iv) less packaged foods. To achieve these aims Singh and Shalini (2016) suggested combining hurdle technologies with predictive microbiology models) and HACCP to achieve food safety and longer shelf lives (Singh and Shalini, 2016).

Vegetable foods such as leafy greens is one example of such a minimally processed food group. The leafy green food chain does not include a pathogen kill step such as boiling, before consumption. This presents an opportunity for pathogens and spoilage bacteria to multiply resulting in food safety risks if consumed, or shortened shelf lives with consequent increasing volumes of food waste. It has been suggested that hurdles or combinations of different preservation methods and Leistner (2000) suggested there were more than 60 potential hurdles such as temperature, acidity (pH), organic acids, or and competitive microorganisms including bacteriophages that could be considered (Leistner, 2000). The preservation methods may reduce bacterial numbers (bacteriocidic) or inhibit growth (bacteriostatic). For example an uninterrupted cold chain could be an helpful hurdle as bacterial growth are limited, while the bacteria contaminating the food before subject to the cold chain would usually remain throughout, and represent a constant risk.

We believe that the leafy green food chain would benefit by combining the HACCP with predictive microbiology and hurdles (combinations of preservation techniques) as a contextual model for improving food safety. However, any combinations of hurdles and the foreseen impact on pathogens should be validated before implemented on an industrial scale. One reason for this need for validation is that the estimates for pathogen reductions on leafy green are very context specific where e.g., species of pathogens might influence the impacts of hurdles. For example *E. coli* O157:H7 survives in acidic environments, but better at lower temperatures (Hsin-Yi and Chou, 2001). Consequently, we agree with (McMeekin et al., 2000) that understanding of physiological processes occurring near the growth/no growth interface is crucial quantification of the impact of hurdles and their intelligent application (McMeekin et al., 2000).

As mentioned previously, the value network of LGV does not start with the processing of leaves but already with the seeds. Use of the hurdle approach in LGV production to date has focused mainly on post-harvest measures, even though most of the contamination risks occur pre-harvest, in primary production (Table 3). It is worth noting that LGV are exposed during the entire value chain to unpredictable environments and that boundary layers are not removed when eaten raw. Thus LGV and their environmental footprint are ingested, which makes the hurdle approach more challenging than that used for other products, e.g., dairy and meat.

**Table 3:** Summary of risk steps in the production of leafy green vegetables where contamination of human pathogens could occur and suggested hurdle options for these.

	**Field/production**	**Harvest**	**Process/wash**	**Pack./storage**	**Distribution**	**Consumer**
Risk steps	Irrigation Soil amendments Plant protection actions Contamination via farm equipment Flooding incidents Heavy rainfall Production location Proximity to animal rearing operation Wild animals Plant damage	Weather conditions at harvest Temperature Machine hygiene Hand harvest hygiene Field containers Irrigation of harvested product to avoid dehydration Cooling of harvested product Plant damage	Wash line hygiene Wash water Employee hygiene Clean containers Storage time before wash Temperature Plant damage Mode of drying the product	Packing line hygieneStorage time before packing Employee hygiene Labeling Type of packaging Temperature Plant damage	Sanitation of vehicles in transport of unpacked product Temperature	Labeling of product (e.g., RTE or not) Food handling Clean utensils Appropriate storage temperature Clean wash water
Organisms	^*^EHEC*, E.coli, Salmonella enterica, Campylobacter, Shigella* spp., *Cyclospora cayatenensis, Chryptosporidium, Yersinia pseudotuberculosis, Listeria* *monocytogenes*					
Hurdle options	Disinfection of irrigation water Fences for wild animals No organic fertilizers	Cooling of produce	Unbroken temperature chain Disinfection of wash water with sanitizing agents Disinfection of wash lines	Unbroken temperature chain Modified atmosphere Relative humidity Disinfection of pack lines	Unbroken temperature chain	

*Blue: Risk steps identified by Gorny et al. (2006)*.

*Green: List of multiple outbreaks on leafy green vegetables reported in at least three regions of the world, including illness and deaths (FAO WHO, 2008)*.

In some cases, more controlled production conditions (soilless production systems) can result in lower initial microbial load. For example, it has been shown that at the end of lettuce shelf-life, soil-grown lettuce can have 1.5 log higher total coliforms than lettuce grown in a soilless system (Selma et al., 2012). In reality, most LGVs are produced in open fields, which means that insights regarding the biology of microbe-microbe and plant-microbe interactions are essential when seeking to determine the route of foodborne pathogen contamination (Critzer and Doyle, 2010) and to develop novel approaches to inhibit or inactivate microbes on LGV.

Possible hurdles for inclusion in the LGV chain could be low temperatures at harvest and along the food chain until consumption (processing, packing, storage, distribution, and pre-consumption), low pH, biopreservation with competitive microflora, aseptic packaging, and washing. The water used for rinsing could be UV-treated and electrolyzed (Rahman et al., 2016) or e.g., chlorine dioxide could be added (Van Haute et al., 2017). By judiciously introducing hurdles such as rinsing and low temperatures (e.g., 0–4 C) at harvest and post-harvest and by using aseptic packaging, a low pathogen and spoilage burden can be maintained and possibly even reduced up to the point of consumption.

Cross-contamination with human enteric pathogens is one of the major issues related to processing of LGV (Allende et al., 2008; Holvoet et al., 2012). Several hurdle options have been explored regarding decontamination of leaves during the washing procedure, with the pathogens commonly considered being *E. coli, Salmonella*, and *L. monocytogenes*. These treatments have led to reductions in pathogens, but none of the methods has proven sufficient to eliminate the pathogenic bacterial load completely. A number of studies have investigated the effect on foodborne pathogens of addition of chlorine to the wash water (Behrsing et al., 2000; Beuchat et al., 2004; Gragg and Brashears, 2010; Davidson et al., 2013; Omac et al., 2015; Pezzuto et al., 2016; Guzel et al., 2017). The efficacy of hypochlorite treatment is dependent on the microbial load of the inoculated pathogen and the target organism (Pezzuto et al. (2016). *Listeria monocytogenes* and *L. innocua* (used as a surrogate for *L. monocytogenes*) have been shown to resist treatment even when hypochlorite is administered in high concentrations, but this treatment is still more successful than washing without an additive (Beuchat et al., 2004; Omac et al., 2015; Guzel et al., 2017). Due to their mode of action (they produce inhibitory compounds at low temperature conditions, but do not grow), lactic acid bacteria are considered plausible candidates in LGV processing. Gragg and Brashears (2010) examined the possibility of using lactic acid bacteria alone, and in combination with a chlorine wash, in order to reduce the total bacterial load of spinach leaves. They found that when inoculated at a density of log 6 CFU/g, the chlorine wash reduced *E. coli* O157:H7 by approximately log 1, the lactic acid bacteria reduced *E. coli* by log 1.4 CFU/g and the multi-hurdle treatment reduced the load by log 1.9 CFU/g.

Although chlorinated water has been proven to be the most efficient sanitizer of LGV, several other have been tested, with varying degrees of efficiency being reported. For example, peroxyacetic acid has been used against *E. coli* O157:H7 and *L. monocytogenes* (Baert et al., 2009) and peracetic acid, percitric acid, sodium bicarbonate and vinegar have been used against *Salmonella* and *Listeria* (Pezzuto et al., 2016). Edible plant extract, such as the pulp and juice of lime fruits and oregano, has been used against *Salmonella, E. coli* O157:H7 and *Shigella sonnei* (Orue et al., 2013).

Vurma et al. (2009) examined the possibility of implementing a sanitizing step already in the cooling process by combining vacuum cooling with gaseous ozone. This multi-hurdle approach resulted in a reduction of log 2.4 CFU/g (initial density log 7CFU/g), but the process damaged the leaves. With an optimized procedure where no leaf damage occurred, the reduction in *E. coli* O157:H7 was log 1.8 CFU/g, which is comparable to that achieved with a chlorine wash (Vurma et al. (2009).

Irradiation with UV-C has antimicrobial effects and is sometimes suggested for use as a surface sterilizer of vegetables and other foodstuff, but also for disinfection of water. Hagele et al. (2016) examined the possibility of using low-dose UV-C irradiation (1.2 kJ/m^2^) on leaf surfaces of endives and for decontamination of the processing water. They found that the reduction in total aerobic bacteria was log 0.9 CFU/g using only UV-C treatment and log 2.1 CFU/g if the UV-C treatment was preceded by washing the endive leaves in warm water (45°C). Treatment of the wash water reduced the total aerobic count in the water by log 1.3 CFU/mL (Hagele et al. (2016).

There are multiple risks of contamination of produce by human enteric pathogens pre-harvest. One of the most common sources of such contamination is irrigation water (Brandl, 2006; Jongman and Korsten, 2017). There are ways of treating the irrigation water prior to use that reduce the bacterial load in the water, such as mechanical filtration using a 0.7 μm filter. This filter size is not sufficient to remove microbial cells, however, although it removes larger particles present in the water that could harbor microorganisms. Another possibility is to use advanced oxidation technology (AOT), where UV-light in combination with a titanium dioxide membrane (TiO_2_) has an oxidative effect on microorganisms, for example *E. coli* and intestinal enterococci. López-Gálvez et al. (2018) treated water used for sprinkler irrigation of baby leaf spinach with chlorine dioxide (ClO_2_; 1–3 mg/L) in a field setting and found that the treatment reduced the load of culturable *E. coli* by log 0.2–0.3 CFU/100 mL water. However, the chlorine dioxide was suspected to have a bacteriostatic effect on the *E. coli*, which caused cells to change into a viable but not culturable state (VBNC). Other factors that are considered to pose a risk of microbial contamination in the pre-harvest phase include contamination via farm equipment, heavy rainfall and flooding incidents, proximity to animal rearing facilities, poor hygiene of workers in the field and poor hygiene of crates and containers used in harvest (Gorny et al., 2006). Presence of wild animals and instances of plant damage by equipment used in the field or by weather events should also be considered. Plant damage can provide potential entry points for internalization of human enteric pathogens (Brandl, 2008; Hartmann et al., 2017). In summary, plant damage can occur at all stages of the production chain, both in the field and in processing and distribution, and it is important to minimize such damage in order to maintain high quality throughout the food chain.

## Conclusions

In order to improve food safety in general, supporting data need to be more easily available to assist primary producers in their decision-making process. There is also a need to develop hygiene criteria to aid validation of proposed interventions. No single risk mitigation measure can achieve food safety for leafy green vegetables. Instead, a combination of mitigation measures, or hurdles, aimed at controlling and/or eliminating pathogens could ensure food safety and quality and freshness. Based on a review of existing literature, it can be concluded that one key element of safe leafy green vegetable production is a low pathogen and spoilage burden pre-harvest. It can also be concluded that the food chain for leafy green vegetables must be a managed in a holistic way for efficient maintenance of food safety and quality. All stakeholders in the food system need to acknowledge their involvement in food safety. Among measures to mitigate the risk of foodborne illnesses being transmitted by produce, avoiding processing practices that involve significant injury to leaves is paramount.

Choice of hurdle must depend on the expected pathogen burden of harvested leafy green vegetables and the food safety objectives at the point of consumption. For example, under EU Regulation 2075/2005, the food safety objective, and consequently the end-product food safety microbiological criterion, for *Salmonella* is absence of the microbe in a 25-g sample of ready-to-eat leafy greens at the point of consumption. Therefore in addition to hurdles applied in the harvest and post-harvest stages of the leafy green vegetable value network, hurdles or risk mitigation measures must be adopted in primary production at all growth stages.

## Author contributions

All authors listed have made a substantial, direct and intellectual contribution to the work, and approved it for publication.

### Conflict of interest statement

The authors declare that the research was conducted in the absence of any commercial or financial relationships that could be construed as a potential conflict of interest.
